# Human platelet lysate as a potential clinical-translatable supplement to support the neurotrophic properties of human adipose-derived stem cells

**DOI:** 10.1186/s13287-020-01949-4

**Published:** 2020-10-06

**Authors:** Silvia Palombella, Martino Guiotto, Gillian C. Higgins, Laurent L. Applegate, Wassim Raffoul, Mario Cherubino, Andrew Hart, Mathis O. Riehle, Pietro G. di Summa

**Affiliations:** 1 Unit of Regenerative Therapy, Service of Plastic, Reconstructive and Hand Surgery, Department of Musculoskeletal Medicine, Lausanne University Hospital, Lausanne, Switzerland; 2 Department of Biotechnology and Life Sciences, University of Insubria, Varese, Italy; 3 Department of Plastic, Reconstructive and Hand Surgery, Centre Hospitalier Universitaire Vaudois (CHUV), Rue du Bugnon, 21, 1011 Lausanne, Switzerland; 4 Centre for Cellular Microenvironment (CeMi), University of Glasgow, Glasgow, UK; 5 Canniesburn Plastic Surgery Unit, Glasgow Royal Infirmary, Glasgow, UK

**Keywords:** Human adipose-derived stem cells (hADSC), Peripheral nerve injury (PNI), Human platelet lysate (hPL), Cell therapy

## Abstract

**Background:**

The autologous nerve graft, despite its donor site morbidity and unpredictable functional recovery, continues to be the gold standard in peripheral nerve repair. Rodent research studies have shown promising results with cell transplantation of human adipose-derived stem cells (hADSC) in a bioengineered conduit, as an alternative strategy for nerve regeneration. To achieve meaningful clinical translation, cell therapy must comply with biosafety. Cell extraction and expansion methods that use animal-derived products, including enzymatic adipose tissue dissociation and the use of fetal bovine serum (FBS) as a culture medium supplement, have the potential for transmission of zoonotic infectious and immunogenicity. Human-platelet-lysate (hPL) serum has been used in recent years in human cell expansion, showing reliability in clinical applications.

**Methods:**

We investigated whether hADSC can be routinely isolated and cultured in a completely xenogeneic-free way (using hPL culture medium supplement and avoiding collagenase digestion) without altering their physiology and stem properties. Outcomes in terms of stem marker expression (CD105, CD90, CD73) and the osteocyte/adipocyte differentiation capacity were compared with classical collagenase digestion and FBS-supplemented hADSC expansion.

**Results:**

We found no significant differences between the two examined extraction and culture protocols in terms of cluster differentiation (CD) marker expression and stem cell plasticity, while hADSC in hPL showed a significantly higher proliferation rate when compared with the usual FBS-added medium. Considering the important key growth factors (particularly brain-derived growth factor (BDNF)) present in hPL, we investigated a possible neurogenic commitment of hADSC when cultured with hPL. Interestingly, hADSC cultured in hPL showed a statistically higher secretion of neurotrophic factors BDNF, glial cell-derived growth factor (GDNF), and nerve-derived growth factor (NFG) than FBS-cultured cells. When cocultured in the presence of primary neurons, hADSC which had been grown under hPL supplementation, showed significantly enhanced neurotrophic properties.

**Conclusions:**

The hPL-supplement medium could improve cell proliferation and neurotropism while maintaining stable cell properties, showing effectiveness in clinical translation and significant potential in peripheral nerve research.

## Background

Peripheral nerve injuries (PNI) can cause motor, sensory, and autonomic disability. Despite the spontaneous regeneration capacity of the peripheral nervous system and microsurgical advancements in nerve repair, the results remain far from optimal, with poor functional outcomes and high patient morbidity [1]. The need to improve the rate of effective regeneration has driven research attention towards cellular therapies, which aim to use autologous therapeutic cells to support the host regeneration process [2].

Stem cells harvested from different adult tissues, including the bone marrow, muscle, and fat, have been used in peripheral nerve regeneration studies, especially in rodent models [3]. In particular, adipose-derived stem cells (hADSC) have attracted interest for clinical application because of benefits including abundance, ease of isolation, high proliferation rate, greater differentiation potential compared to other mesenchymal stem cells (MSCs), and their immunomodulatory and angiogenic properties [4–6]. For all these reasons, hADSC are a good candidate for cell-based therapies and could play a promising role in regenerative medicine and nerve tissue engineering [7].

Stem cell therapy is subjected to safety concerns, and the potential for zoonotic transmission during the use of animal-derived products in cell culture is a serious consideration [8, 9]. The manipulation with xenogeneic components and in particular, the common use of fetal bovine serum (FBS) as a growth factor supplement for cell culture, could increase the risk of immune reactivity in the host patient and exposure to viral, bacterial, or prion infection [10, 11]. Immune reaction and diffuse urticariform development have been described in individuals who received several doses of MSCs produced with FBS [12, 13]. Moreover, FBS is subjected to batch-to-batch variability, finally impacting on data reproducibility. The avoidance of chemical tissue dissociation, the definition of more reliable and reproducible methods, and the substitution of FBS with a supplement free of xenogeneic proteins are essential steps to ensure cell usage in accordance with good manufacturing practice (GMP) guidelines [9, 14].

Human platelet lysate (hPL) was suggested as a substitute to FBS since it can be easily obtained, as pooled blood, in large quantitates from apheresis products and buffy coats [15].

Previous studies showed that compared with FBS, the hPL supplement supports cell viability, enhances proliferation, delays senescence, ensures cell genomic stability, and preserves cellular immunophenotype, even in advanced cell passages [12].

Despite the growing use of hPL in vitro cell culture and the urgent interest in translational purposes, the application of hPL needs to be defined properly on quality issues and safety criteria (e.g., open questions such as virus inactivation or prion transmission) [16, 17]. These steps are essential, focusing on the differences between the manufacturing and clinical implications of hPL and the traditional fields of transfusion medicine. Moreover, the hPL application involves manufacturers, distributors, or users who may not have the complete set of knowledge on the quality and safety criteria of blood products necessary to guarantee the GMP compliance [16].

Nevertheless, the complete set of platelet-released factors has not been described yet. hPL is known for containing a wide variety of growth factors, including neurotrophin 3 (NTF3), nerve growth factor (NGF), brain-derived growth factor (BDNF), and glial cell line-derived neurotrophic factor (GDNF). These molecules are involved in Schwann cells (SCs) survival, differentiation, release of extra-cellular matrix (ECM) molecules, and trophic factors and provide a permissive environment for axonal elongation [18, 19].

Taking these considerations into account, the aim of this work is to verify if hADSC could be routinely cultured in a completely xenogeneic-free way, without altering their physiology and stemness compared to classical FBS-cultured hADSC. Moreover, we investigated the potential of hPL in maintaining and eventually enhancing neurogenic properties of hADSC for peripheral nerve regeneration.

## Materials and methods

### Experimental design

hADSC were isolated from abdominal adipose tissue of five healthy women (average age 49 ± 2) who underwent breast reconstruction using abdominal autologous flaps (deep inferior epigastric perforator flaps, DIEP) at the University Hospital of Lausanne, CHUV, Lausanne, Switzerland, and at the Canniesburn Plastic Surgery Unit, Glasgow Royal Infirmary, Glasgow, Scotland. The discarded part of the flap and the adipose tissue were obtained after patients signed informed consent. All protocols were reviewed and approved by the hospital, local BioBank (number 314 GGC) and University (UofG) ethics committees in accordance with the Declaration of Helsinki.

In order to evaluate if eventual differences were due only to the medium supplement (hPL or FBS) or to the isolation method (collagenase or explant), we established four conditions:

- Collagenase + FBS
- Collagenase + hPL
- Explant + FBS
- Explant + hPL

The adipose tissue obtained from the same patient was used to establish both explant and single-cell cultures after enzymatic digestion by collagenase. hADSC isolated with either method were cultured in parallel in D-MEM (Gibco, Paisley, UK) supplemented with 10% FBS (Sigma Aldrich, Zofingen, Switzerland) or 5% heparin-free hPL (Human Platelet Lysate FD (GMP Grade) Catalog No. ABIN6720615, Antibodies, Aachen, Germany). HPL was manufactured from platelet units obtained from 150 to 300 healthy blood donors at FDA-licensed blood centers. Platelets were activated by freezing and thawing them three times, the cell debris is centrifuged off, and the supernatant with the cytokines and growth factors are processed further. Fibrinogen was depleted. FBS containing medium was supplemented with 2 mM l-glutamine (Gibco). No antibiotics were used in any culture media, as per GMP directions regarding the use of cell products for human use. When cells reached 70–80% confluence, they were detached using an animal-free trypsin substitute, TrypLe (Gibco) as indicated by the manufacturer.

### hADSC isolation and culture

The tissue sample was initially washed twice with PBS (Gibco) to remove the excess blood and divided equally into two parts: one for enzymatic digestion and one to establish explant cultures. For collagenase digestion, the adipose tissue was mechanically minced with scissors/scalpel and enzymatically dissociated with 0.15% (w/v) collagenase II (Gibco, Grand Island, USA) at 37 °C for 1 h in a water bath. The resulting cell solution was filtered through a 100-μm filter to remove undissociated tissue before stopping the collagenase reaction with one volume of the corresponding complete medium with FBS or hPL. After centrifugation at 1075 relative centrifugal force (RCF) for 10 min, the stromal vascular fraction was resuspended in 5 mL of the corresponding complete medium, plated in T25 flasks, and cultured at 37 °C and 5% CO_2_. The medium was changed after 24 h to remove erythrocytes. On the other hand, the tissue biopsy was minced and microdissected (diameter < 5 mm for single piece), and approximately 1 g of tissue was mechanically dispersed in a 10-cm Petri dish. hADSC were selected thanks to their plastic adherence without enzymatic digestion [20]. The medium was changed every 3–4 days until cell confluence for all culture conditions. All experiments were performed on cells at passages 2–4 (P2–P4) in order to reduce the selection of hADSC subpopulations. Cell morphology (three technical and biological (3 donors) repeats) in the four conditions was monitored daily under phase-contrast microscope. (10x, Olympus IX81, Hamburg, Germany).

### Characterization of hADSC by flow cytometry

To evaluate immunophenotype, hADSC cultured in the four conditions (passage 2 (P2)) were stained with the following antibodies: anti-CD73 (1:1000, IgG1, BD Biosciences, monoclonal, FITC-labeled), anti-CD90 (1:1000, IgG1, BD Biosciences, monoclonal, FITC-labeled), anti-CD105 (1:200, IgG1, BD Biosciences, monoclonal, PE-labeled) as positive mesenchymal stem cell markers; anti-CD34 (1:66, IgG1, BD Biosciences, monoclonal, FITC-labeled); and anti-CD45 (1:66, IgG1, BD Biosciences, monoclonal, FITC-labeled), as negative endothelial and hemopoietic markers, respectively. Appropriate isotype controls were used for each fluorophore (1:1000, IgG1, BD Biosciences, monoclonal, FITC-labeled; 1:50, IgG1, BD Biosciences, monoclonal, PE-labeled; 1:50, IgG2b, BD Biosciences, monoclonal, FITC-labeled). For each antigen, approximately 100,000 cells were resuspended in 100 μL of antibody diluted in flow cytometry buffer (0.2% FBS in PBS) and incubated for 1 h, agitated in the dark. The cell surface phenotype was subsequently assessed by BD Accuri C6 apparatus (Erembodegem, Belgium). Experiments were conducted in technical and biological triplicates.

### Proliferation of hADSC

The proliferation of hADSC cultured in the four conditions was assessed daily for 7 days with the CellTiter 96® AQueous One Solution Cell Proliferation Assay (Promega, Zurich, Switzerland) according to the manufacturer’s instruction. Briefly, 5 × 10^2^ cells (P2, P3, P4) per well were seeded into 96-well plates and cultured at 37 °C 5% CO_2_. The day of the test the medium was replaced with 100 μL of fresh medium added with 20 μL of assay reagent per well. To allow the development of the reaction, hADSC were incubated for two and half hours before reading absorbance at 490 nm with an infinite F50 spectrophotometer (Tecan, Mannerdorf, Switzerland). Experiments were conducted in technical and biological triplicates.

### hADSC differentiation potential

To compare the multipotency of FBS-cultured with hPL-cultured hADSC, cells were differentiated into adipocytes and osteocytes. For both differentiation protocols, about 5000 cells (P3) were seeded in 12-well plates and maintained in differentiation medium for 2 weeks with the medium changed every 2 days. Adipogenic differentiation medium was composed of complete medium added with 1 μM dexamethasone (Sigma Aldrich), 100 μM indomethacin (Sigma Aldrich), 100 μM 3-isobutyl-1-methylxantine (Sigma Aldrich), and 1x ITS (Sigma Aldrich). The osteogenic differentiation medium was composed of complete medium added with 50 μg/mL ascorbic acid (Sigma Aldrich), 5 mM β-glycerophosphate (Sigma Aldrich), and 100 nM dexamethasone (Sigma Aldrich). Cells cultured with the corresponding complete medium without differentiation factors were used as a negative control. After the differentiation period, hADSC were fixed with 4% paraformaldehyde for 10 min at room temperature and stained with Oil Red O (0.18%, Sigma Aldrich) for fat droplets evaluation or Alizarin Red (0.5%, Sigma Aldrich) to detect calcium deposits. Images were acquired with an optical microscope in bright field (IX81, Olympus, Iowa, USA). Experiments (adipogenic) were conducted in technical and biological triplicates, while the osteogenic one in three technical repeats and five biological repeats (5 donors)).

### Evaluation of secreted factors

The conditioned medium was collected when hADSC reached confluence and centrifuged at 4300 relative centrifugal force (RCF) for 10 min to remove cellular debris, and supernatants were transferred to a new tube and stored at − 20 °C until processing. Fresh medium non-conditioned was used as a negative control. Before proceeding with NGF and GDNF evaluation, samples were concentrated using filter columns with a 30-kDa cutoff (Millipore Amicon ultra-4 centrifugal filter devices). The stored medium was centrifuged at 4300 RCF for 10 min at room temperature. Concentrated samples were brought up to a minimum volume sufficient for ELISA, obtaining a final concentration factor of 8×. Subsequently, samples were analyzed with ELISA kits for BDNF (Millipore, Schaffhausen, Switzerland), NGF (Abcam, Basel, Switzerland), and GDNF (Thermo Fischer Scientific, Loughborough, UK), according to the manufacturer’s instruction. Each sample was assayed in duplicate, and the absorbance determined at 450 nm with an infinite F50 spectrophotometer (Tecan Group, Männedorf, Switzerland). The quantity of secreted factors was calculated from standard curves produced using recombinant proteins, multiplied for dilution factor, and normalized for 1 × 10^6^ hADSC, in order to eliminate differences due to variation in proliferation rate between samples. Experiments were conducted in technical and biological triplicates.

### RNA isolation and retrotranscription

In order to assess gene expression of SC markers, the total RNA was extracted with TRIzol Reagent (Invitrogen, Basel, Switzerland) following manufacturer instructions. Briefly, about 1 × 10^6^ hADSC (cultured in classical (collagenase + FBS) and cell therapy-ready (explant + hPL) conditions) were resuspended in 1 mL TRIzol Reagent, incubated 5 min at room temperature, and after adding 200 μL of chloroform, the samples were centrifuged for 15 min at 12900 RCF at 4 °C. The resulting aqueous phase was transferred into a new tube, and RNA was precipitated by adding 500 μL of isopropanol, incubation 10 min at room temperature, and centrifugation 10 min at 12900 RCF at 4 °C. The washing step consisted of 1 mL of 75% ethanol and centrifugation 5 min at 8062 RCF at 4 °C. After drying, the RNA pellet was resuspended in 20–50 μL water depending on size. RNA concentration was determined using NanoDrop N-100 (Thermo Fisher Scientific).

The first-strand cDNA was synthesized using the TaqMan Reverse Transcription Reagents (Applied Biosystems, Rotkreuz, Switzerland) according to the manufacturer’s instructions. Retrotranscription was based on random hexamers used at a final concentration of 2.5 μM and the thermocycler set up as follows: 25 °C for 10 min, 37 °C for 30 min, and 95 °C for 5 min. One microgram of total RNA was used as an initial template in a final reaction volume of 20 μL, and each sample was subsequently diluted to the final concentration of 5 ng/μL.

### Quantitative PCR

qPCR (samples from both classical and cell therapy-ready) was executed using a StepOnePlus Real-Time PCR system (Applied Biosystems). The reactions were carried out in triplicates using the Fast SYBR Green Master Mix (Applied Biosystems). Each reaction was performed using 10 ng starting cDNA in a final volume of 20 μL; primers were used at a final concentration of 300 nM each. The thermocycler program had an initial hot start step at 95 °C for 20 s, followed by 40 cycles at 95 °C for 3 s and 60 °C for 30 s. To confirm primer specificity, a melting curve analysis was performed after each amplification. Primer sequences are reported in Table 1. Reference genes were selected as described previously [21]. The expression of target genes was expressed as 2^-ΔΔCt^ compared to the control sample of classic culture [22]. Experiments were conducted in technical and biological triplicates.

**Table 1:** Polymerase chain reaction gene analysis. Primer sequences of the genes used to evaluate human adipose stem cells neurogenic commitment

Gene	Primer sequence (5′ ≥ 3′)	Amplicon length (bp)	Accession number
**RPL13a**	Fw: TATGAGTGAAAGGGAGCC Rv: ATGACCAGGTGGAAAGTC	82	NM_001270491.1
**RPS18**	Fw: GAGGTGGAACGTGTGATC Rv: GGACCTGGCTGTATTTTC	109	NM_022551.2
**UBC**	Fw: CACTGGCAAGACCATCACC Rv: TCAACCTCTGCTGGTCAGG	107	NM_021009.6
**NTRK1**	Fw: GCCACATCATCGAGAACCCA Rv: CTCCCACTTGAGCACGATGT	83	NM_001012331.1
**NTRK2**	Fw: TCTGCTCACTTCATGGGCTG Rv: GTGGTGTCCCCGATGTCATT	110	NM_006180.4
**NGFR**	Fw: CTGAGGCACCTCCAGAACAA Rv: ACAGGGATGAGGTTGTCGGT	118	NM_002507.3
**GFRA1**	Fw: CCACTCATGTTTTGCCACCG Rv: ACAGAGGTGTGTATTGCCCG	80	NM_005264.5

### Immunofluorescence

About 10,000 cells/cm^2^ (hADSC cultured in classical (collagenase + FBS) and cell therapy-ready (explant + hPL) conditions) were cultured in chamber slides (Ibidi, Baar, Switzerland) for 24 h before fixation with 4% paraformaldehyde at room temperature for 20 min. Samples were blocked in 2% BSA (Sigma Aldrich) in PBS and permeabilized with 0.1% Triton-X100 (AppliChem, Darmstadt, Germany) for cytosolic/nuclear antigens or with 0.1% Tween 20 (AppliChem) for surface antigens. After appropriate permeabilization, cells were incubated with the primary antibody anti-STRO-1 (1:400, Thermo Fischer Scientific, mouse monoclonal), anti-NES (1:200, Abcam, rabbit monoclonal), anti-GFAP (1:200, Abcam, rabbit monoclonal), and anti-MPZ (1:100, Abcam, rabbit polyclonal) at 4 °C. The following day, slides were incubated for 1 h at room temperature with FITC-conjugated secondary antibody (anti-mouse, 1:200, Abcam, polyclonal). Cell nuclei were labeled with Hoechst 33342 (Thermo Fischer, 20 mM) added to the secondary antibody solution at a concentration of 8 μM. Samples were examined under a fluorescence microscope at ×10 magnification (Olympus IX81, HamBurg, Germany). Experiments were conducted in technical and biological triplicates.

### Functional cocultures with primary neurons

Neonatal Sprague Dawley rats (1–3 days old) dissection and Dorsal Root Ganglia (DRG) extraction were performed according to previous protocols [23]. DRG organotypic explants after delicate microsurgical root cleaning were immediately cultured (a single one for each well) according to the following conditions:

- Condition 1 (control): single DRG grown on poly-l-lysine (PLL)-coated coverslip for 48 h at 37 °C, 5% CO^2^ in 200 μL of L-15 media (Sigma-Aldrich), 50 μg/ml *N*-acetyl-cysteine (NAC, Sigma-Aldrich), 1% penicillin-streptomycin (Pen-S, GE Healthcare), 10 ng/ml NGF 2.5S (NGF, Invitrogen), and 5% human platelet lysate (hPL, Antibodies) or 10% fetal bovine serum (FBS, Sigma Aldrich).
- Condition 2 (direct co-cultures hPL/FBS): single DRG grown in contact with hADSC (15,000/cm^2^ pre-expanded for 48 h with D-MEM supplemented with either 5% hPL or 10% FBS) on PLL-coated coverslip for 48 h at 37 °C, 5% CO^2^ in 100 μL of L-15 media, 50 μg/ml NAC, 1% Pen-S, 10 ng/ml NGF, and 100 μL of D-MEM with or without 5% hPL or 10% FBS.
- Condition 3 (indirect co-cultures hPL/FBS): single DRG grown on PLL-coated coverslip for 48 h at 37 °C, 5% CO^2^ in 100 μL of L-15 media, 50 μg/ml NAC, 1% Pen-S, 10 ng/ml NGF, and 100 μL of either hPL-hADSC-conditioned medium (hADSC pre-expanded for 48 h in D-MEM with 5% hPL) or FBS-hADSC-conditioned medium (hADSC pre-expanded for 48 h in D-MEM with 10% FBS.)

After 48 h, DRGs were fixed and stained with mouse anti-ß3-tubulin (Sigma Aldrich TubIII, 1:100) and FITC-coupled phalloidin (Thermo Fischer; 1:200) followed by Texas-red labeled anti-mouse (1:100, Vector Laboratories, USA) and DAPI (Vecta-Shield, Vector Laboratories, Peterborough UK). Immunofluorescence images were acquired as described previously [23]. DRG scanning image was analyzed in ImageJ Fiji software [24]. Results were expressed as the maximal neurite length and neurite extension area. The neurite length was manually evaluated using the freehand line tool after setting the appropriate scale for calibration. For every picture, at least 5 different measurements were performed to identify the longest neurite. The area was identified with the freehand area tool following the perimeter of each neurite and removing the area of the DRG body. Experiments were conducted in biological triplicates (3 hADSC donors) and 9 technical repeats.

### Statistical analysis

All data were expressed as average ± standard error (SE) of the mean for at least three technical repeats and three biological repeats. One-way analysis of variance (ANOVA) with Tukey’s multiple comparison test was used to assess statistical significance among three or more groups. Student *t* test was used to test the hypothesis about the means of two groups when data were normally distributed; otherwise, Mann-Whitney was applied. To verify the data spread, D’Agostino and Pearson omnibus normality test was applied.

Significance was expressed as *p < 0.05, **p < 0.01, and ***p < 0.001. All analysis was performed using GraphPad Prism 6 for Mac (GraphPad Software, La Jolla California USA).

## Results

### Immunophenotype of hADSC is mostly influenced by the serum supplement used

In order to investigate if hADSC phenotype, morphology, or behavior were dependent on the isolation method or culture serum, cells were isolated and cultured according to these four different conditions: (1) collagenase + FBS, (2) collagenase + hPL, (3) explant + FBS, and (4) explant + hPL. The typical characterization of stem cells was subsequently performed.

Variations of hADSC immunophenotype were evaluated by flow cytometry. In general, hADSC cultured in all conditions lacked the expression (< 2%) of CD34 and CD45, indicating that cell cultures lacked hematopoietic stem cells or their derivatives. Regardless of culture condition, the majority of hADSC were positive (> 90%) for CD105. The expression of CD73 on the hADSC was independent of the culture additive or isolation method. Conversely, there was however a significant difference in the expression of CD90. In the FBS-cultured cells, there was 76% and 58% expression of CD90 with collagenase and explant method respectively; with hPL supplementation, there was 95% CD90 expression with both collagenase and explant methods (Table 2). These data suggest not only that the supplement used can influence the expression of typical stemness markers, but also that it can sustain a higher number of hADSC for a longer period of time (e.g., hPL). Overall, the isolation method had no significant influence on cell population immunophenotype.

**Table 2:** Immunophenotype of hADSC. Hemopoietic (CD34) and endothelial (CD45) markers were negative in all culture conditions (< 2%). The MSC markers CD73 and CD90 were expressed (> 90%) in hPL-cultured cells without statistical differences among groups. CD105 was expressed in all conditions (> 90%). The isolation method did not influence marker expression. Experiments were conducted in three technical and biological (3 donors) repeats

Protein marker	Condition 1 (collagenase + FBS) (%)	Condition 2 (explant + FBS) (%)	Condition 3 (collagenase + HPL) (%)	Condition 4 (explant + HPL) (%)
**CD34**	0.86 ± 0.16	1.02 ± 0.05	1.04 ± 0.20	0.96 ± 0.05
**CD45**	1.78 ± 0.91	1.06 ± 0.06	1.45 ± 0.59	0.88 ± 0.08
**CD73**	86.75 ± 6.30	87.43 ± 8.16	96.76 ± 2.84	97.95 ± 1.00
**CD90**	76.19 ± 11.66	58.38 ± 14.61	97.97 ± 1.39	96.33 ± 3.04
**CD105**	96.42 ± 1.00	95.89 ± 1.50	97.23 ± 0.98	97.91 ± 1.44

### hPL enhances hADSC proliferation

**Fig. 1:**
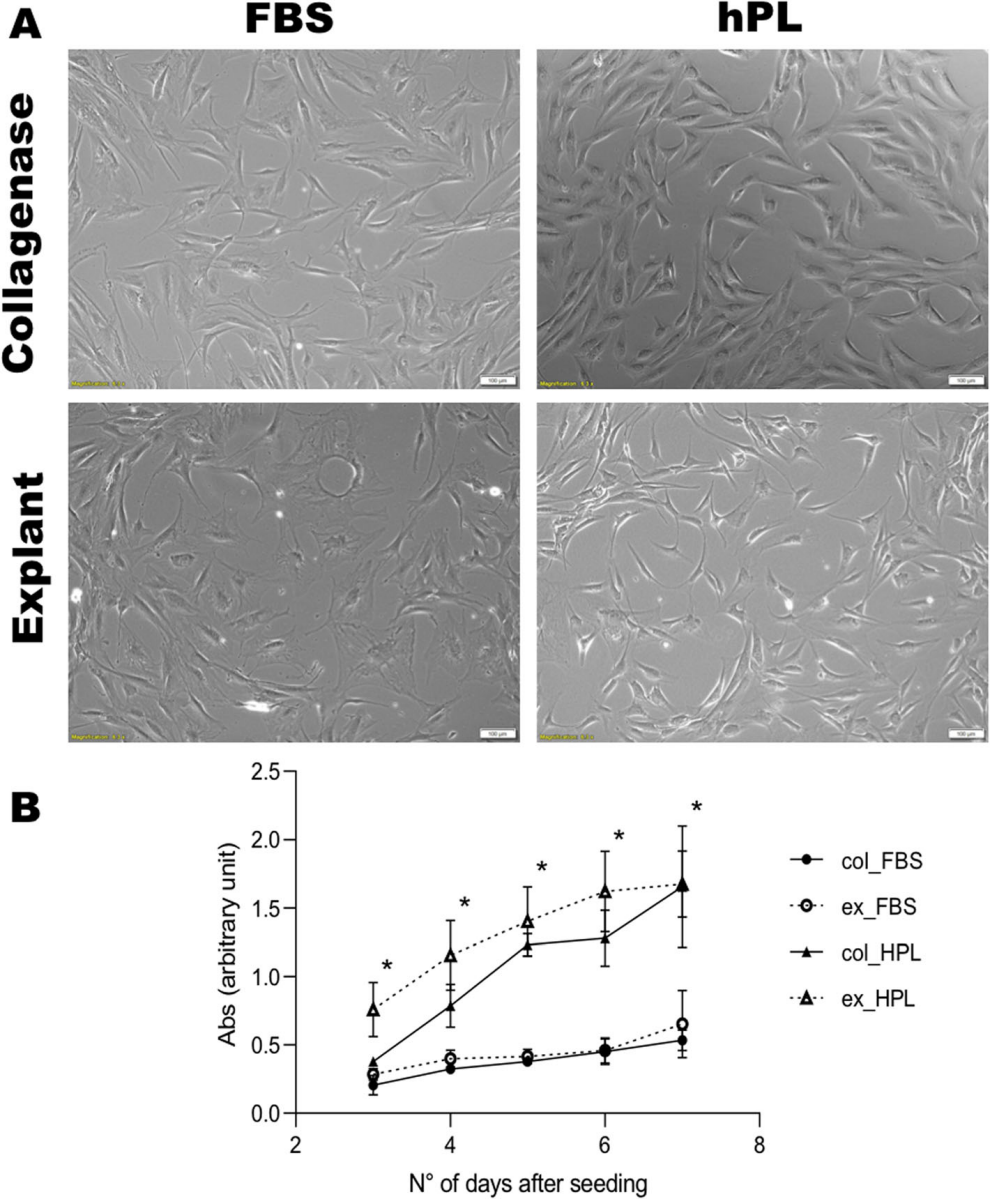
Morphology and proliferation of hADSC in the four different conditions. **a** Representative phase-contrast pictures of hADSC cultured with FBS (left column) or hPL (right column) after isolation with collagenase (upper row) or with explant method (lower row). **b** Cell proliferation was evaluated measuring metabolic activity of hADSC from days 3 to 7 after seeding. Experiments were conducted in three technical and biological (3 donors) repeats. One-way ANOVA multiple comparisons tests were used for proliferation assays to assess statistical significance among the four examined extraction/culture conditions: (*p* < 0.05)

Independent of the isolation method (collagenase or explant), hADSC cultured with FBS showed classic fibroblast-like morphology (Fig. 1a). Conversely, hADSC cultured with hPL appeared a less flatted and more elongated, spindle-shaped compared to FBS-cultured hADSC (Fig. 1a), suggesting that morphology was influenced by culture serum.

Cell proliferation was evaluated measuring the metabolic activity of hADSC from day 3 to 7 after seeding. As reported in Fig. 1b, the proliferation of hADSC cultured in condition 2 (collagenase + hPL) was significantly higher than measured for the control group (classical culture condition) at the same time point. The proliferation of hADSC cultured under “cell therapy-ready” conditions (explant + hPL) was significantly higher compared to hADSC cultured under classical culture condition (collagenase + FBS) at 6 days after seeding. Moreover, no significant differences in cell proliferation were observed comparing the two cell isolation methods, in line with the rest of our data, that the isolation method was not an influencing factor.

### hADSC multipotency is retained when cultured with hPL

**Fig. 2:**
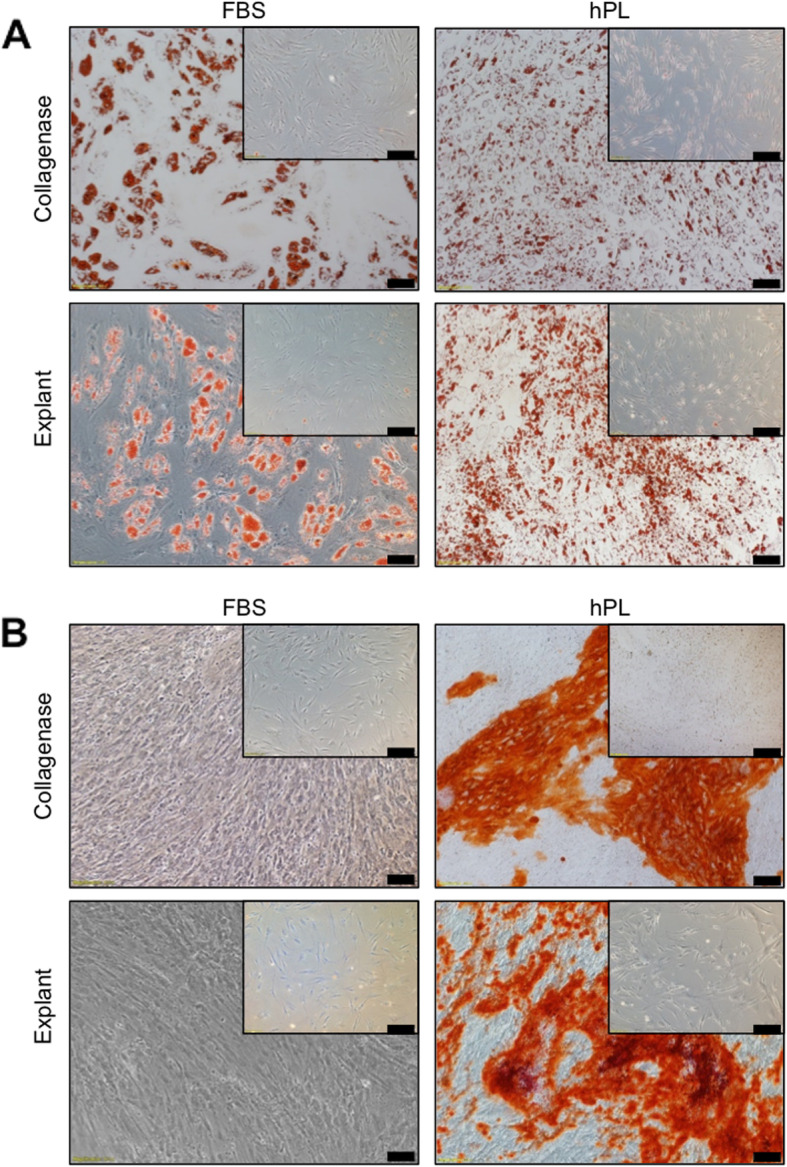
Differentiation of hADSC in the four different conditions. **a** Pictures representing the good adipogenic differentiation rate of hADSC cultured in all conditions. **b** Osteogenic differentiation of hADSC occurred only with hPL-based cultures (right panels) but not with FBS cultures (left panels). Insets show control cells without differentiation medium. Black scale bars indicate 100 μm. Experiments were conducted in three technical and three biological (3 donors) repeats for adipogenic differentiation while in 5 biological replicates for the osteogenic one

hADSC obtained from all culture conditions were differentiated into adipocytes and osteocytes to assess their multipotency. Adipogenic differentiation was achieved by hADSC in all examined conditions (Fig. 2a). However, from a qualitative point of view, hPL-cultured cells (Fig. 2a, right panel) showed a more pronounced differentiation rate, with smaller cells compared to FBS-cultured cells (Fig. 2a, left panels). Control cells not incubated with differentiation medium showed no Oil-red-O staining, even though some deposited lipid residuals from hPL were stained (Fig. 2a, insets).

Osteogenic differentiation was evaluated with Alizarin Red to identify calcium-containing ECM and cells. The staining evidenced that hADSC cultured with differentiation media containing FBS were not able to differentiate, and no calcium deposits were detected, partly supporting the low expression of CD90 [25] in FBS-cultured cells previously detected (Fig. 2b, left panels). On the contrary, hPL-cultured cells were stained in red indicating cell differentiation towards an osteocyte phenotype (Fig. 2b, right panels). Control cells cultured without differentiation factors showed no Alizarin red staining (Fig. 2b, insets).

### hPL greatly increases secretion of neurotrophic growth factors

**Fig. 3:**
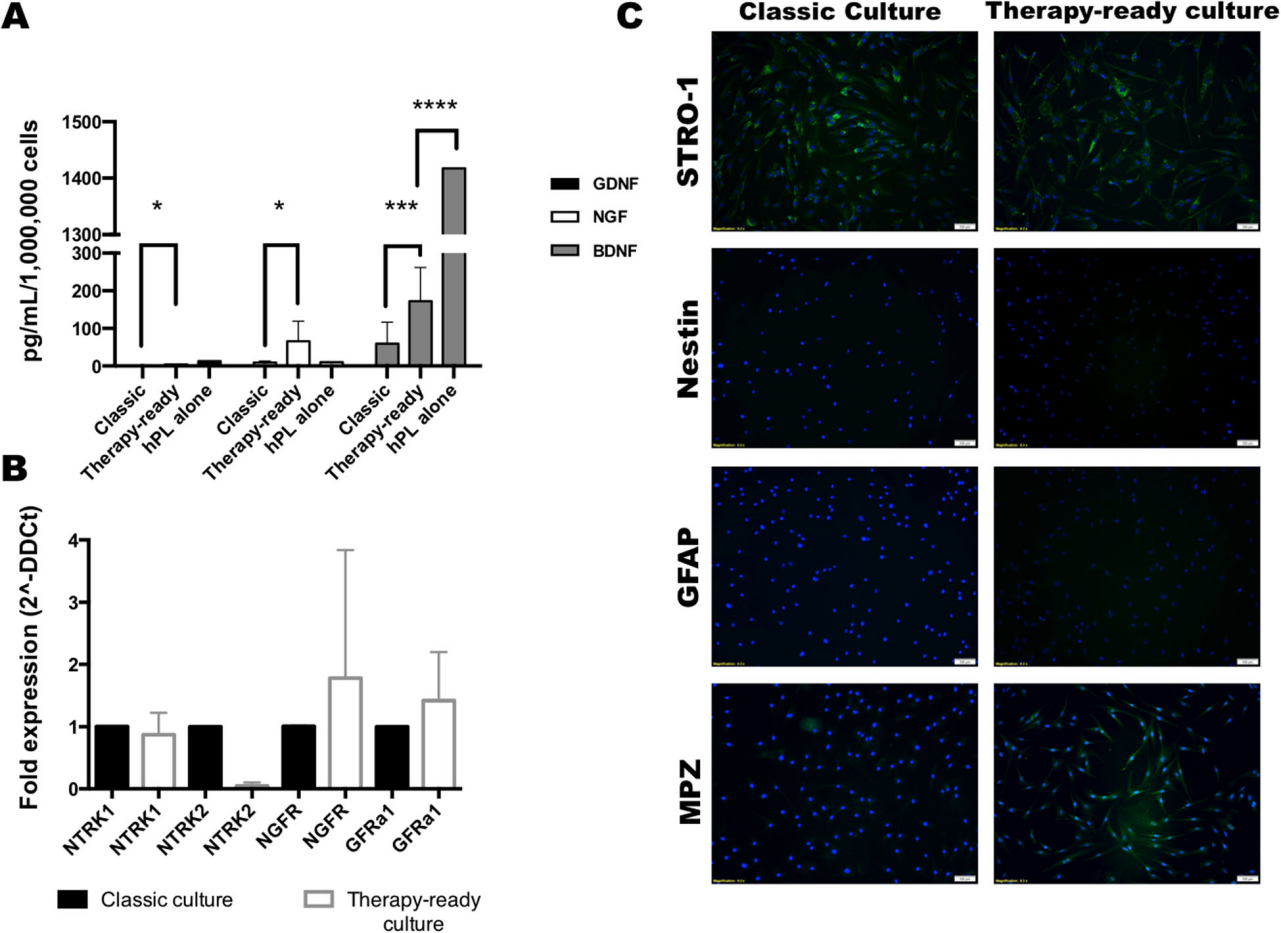
Evaluation of SC markers in hADSC in the two conditions. **a** Evaluation of secreted factors (NGF, BDNF, and GDNF) by hADSC cultured in classical and cell therapy-ready conditions. One-way ANOVA multiple comparisons tests were used for assessing statistical significance among the examined conditions: (*p < 0.05, **p < 0.01, ***p < 0.001, ****p < 0.0001). **b** Gene expression of hADSC surface receptors, *NTRK1* (coding for the high-affinity receptor for NGF), *NTRK2* (coding for the high-affinity receptor for BDNF), *NGFR* (coding for the low-affinity receptor for NGF and BDNF), and *GFRA1* (coding for the receptor for GDNF). **c** Representative fluorescence pictures of intracellular spotted signal detection of STRO-1, Nestin, GFAP, and MPZ in hADSC cultured in classical (left panel) and cell therapy-ready conditions (right panel). Experiments were conducted in three technical and biological (3 donors) repeats

Since the above-outlined data showed that differences in the hADSC phenotype attained under both differentiating conditions were influenced only by the culture supplement (FBS or hPL) and not by the isolation method (collagenase or explant), subsequent experiments to evaluate neurotrophic features were therefore performed only on hADSC cultured in classical (collagenase + FBS) and cell therapy-ready (explant + hPL) conditions. In particular, we evaluated the secretion of main neurotrophic GFs (GDNF, NGF, and BDNF) in conditioned medium collected when cells reached confluence. Normal media with either FBS or hPL were used to evaluate the background levels of these GFs. Interestingly, therapy-ready-cultured cells secreted all the investigated growth factors statistically more than classic-cultured cells, suggesting that hPL can increase neurogenic properties of hADSC. GDNF was not secreted by hADSC cultured in classic conditions (FBS: 0 pg/mL/1000,000 cells); instead, it was secreted four times more from cells cultured in therapy-ready conditions (hPL: 4.03 ± 0.43 pg/mL/1000,000 cells, *p* < 0.05). Besides that, the secretion of NGF was increased almost three times when hADSC were cultured with hPL (12.24 ± 1.31 pg/mL/1000,000 cells, *p* < 0.05) compared to FBS (4.91 ± 0.75 pg/mL/1000,000 cells). The BDNF in conditioned medium evidenced the following values FBS: 2.09 ± 2.09 pg/mL/1000,000 cells and hPL: 82.42 ± 0.81 pg/mL/1000,000 cells, (*p* < 0.001). Surprisingly, the level of BDNF secreted from hPL-cultured cells was greatly higher than FBS-cultured cells, but lower than hPL alone (hPL not conditioned medium: BDNF 1417.31 pg/mL, *p* < 0.0001) (Fig. 3a). Noteworthy, all the other investigated factors were already present in hPL not conditioned medium, in a not significantly different concentration compared to hADSC conditioned medium (GDNF 12.54 pg/mL and NGF 10.01 pg/mL). Since we used human-specific antibodies, these proteins were not detected in FBS-added medium.

### Growth factors contained in hPL can influence the expression of cell own receptors on hADSC surface

Considering the high amount of neurotrophic growth factors contained in hPL, we investigated the expression of hADSC surface receptors, namely *NTRK1* (coding for the high-affinity receptor for NGF), *NTRK2* (coding for the high-affinity receptor for BDNF), *NGFR* (coding for the low-affinity receptor for NGF and BDNF), and *GFRA1* (coding for the receptor for GDNF). Reference gene analysis evidenced that three genes were enough to correctly normalize the expression of target genes. In particular, the genes stably expressed between FBS- and hPL-cultured hADSC for all donors were *RPS18*, *UBC*, and *RPL13a*.

The most striking result was that the expression of *NTRK2* was virtually nothing in therapy ready-cultured hADSC compared to classical culture conditions (fold times 0.05 ± 0.03), suggesting a strong effect exerted by the high amount of BDNF contained in the hPL-supplemented medium. Conversely, concerning the expression of *NGFR* and *GFRA1*, the amount of their mRNA was not significantly changed, despite a slight increase (*NGFR*: fold times 1.78 ± 1.19; *GFRA1*: fold times 1.42 ± 0.45). Similarly, the expression of *NTRK1* was not affected by different culture conditions (fold time 0.871 ± 0.2), suggesting that the amount of NGF and GDNF contained in hPL-supplemented medium were not enough to modify the expression of their own receptors (Fig. 3b).

### hADSC cultured with hPL are not neural committed

Since the hPL supplement included a high amount of critical neurotrophic growth factors, we verified if hADSC could have been neurally committed during in vitro culture. In particular, we considered three neural markers: nestin (indicating neural precursor cells), GFAP (mature astrocyte marker), and MPZ (Schwann cell-specific marker). In all investigated samples, we did not detect the signal of nestin and GFAP, suggesting that hADSC were not SC-like committed. On the contrary, some hPL-cultured hADSC showed a low level of MPZ (Fig. 3c).

### hPL-treated hADSC enhance neurite outgrowth in functional co-cultures with primary neurons

**Fig. 4:**
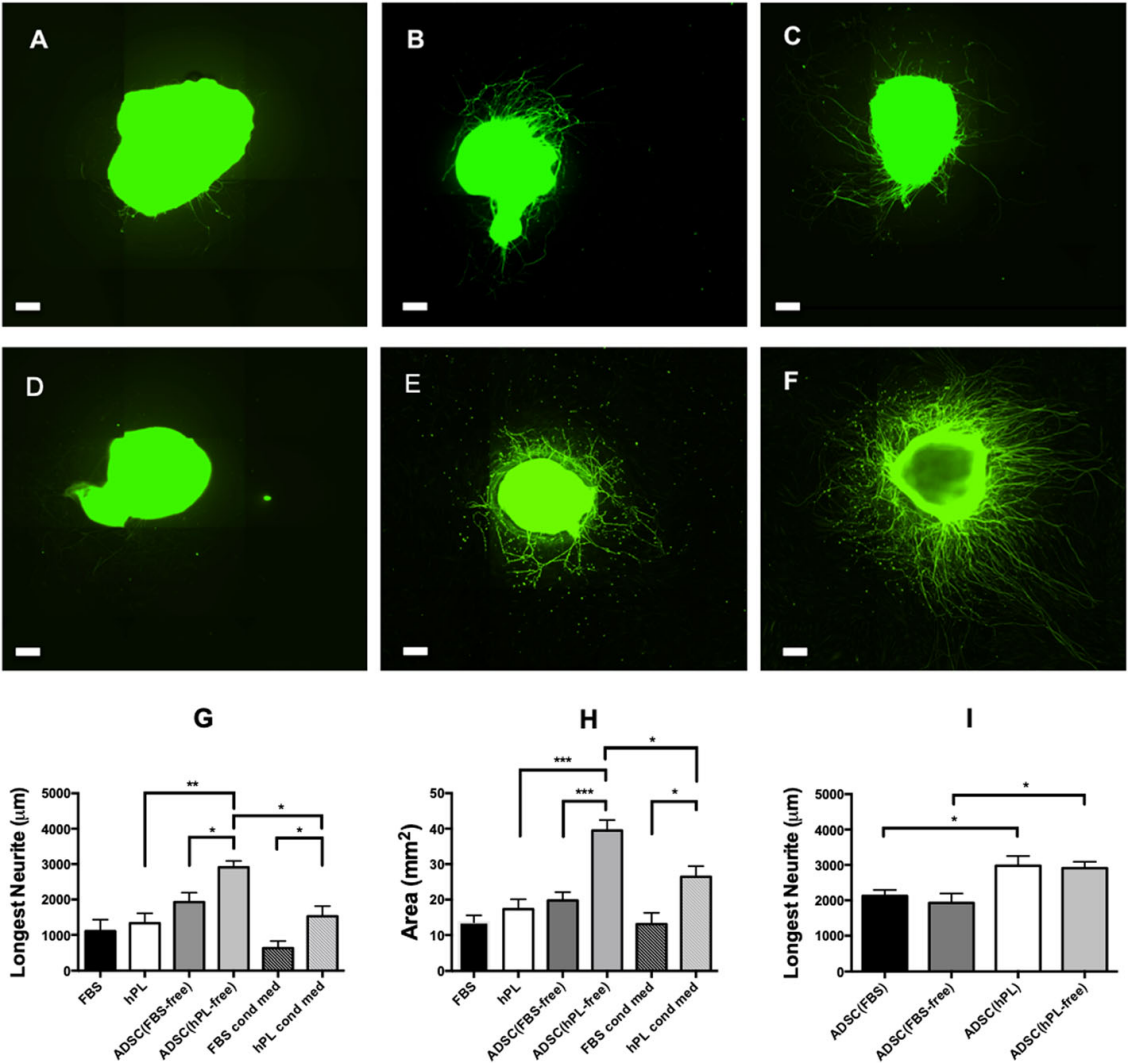
Functional analysis using an in vitro co-culture model with DRG. Immunofluorescence microscopy (anti-ß3-Tubulin) of DRG alone (**a** FBS, **d** hPL), indirect co-culture (**b** FBS cond med, **e** hPL cond med), and direct co-culture serum-free (**c** ADSC (FBS-free), **f** ADSC (hPL-free)). Neurite maximal length (**g**) and neurite area (**h**) of single DRG grown alone, in direct or indirect co-culture with hADSC expanded in FBS or hPL. **i** Neurite maximal length of direct co-culture of single DRG with hADSC expanded in FBS or hPL—comparison between no serum-free and serum-free co-cultures. Scale bar: 500 μm. Experiments were conducted in biological triplicates (3 hADSC donors) and 9 technical repeats one-way ANOVA with Tukey’s multiple comparison tests were used for assessing statistical significance among the examined conditions: (*p < 0.05, **p < 0.01, ***p < 0.001, ****p < 0.0001)

To understand the impact of hPL on DRG outgrowth, single DRGs were cultured with their medium supplemented by either 10% FBS or 5% hPL. Despite the higher concentration of trophic factors of the platelet lysate, especially neural growth factors, no significant difference was found for the outgrowth between the two media additives (FBS, hPL) according to the two parameters analyzed (neurite length and area). This suggests that these sera alone could not significantly impact on the regeneration of sensory neurons (Fig. 4g, h).

To assess if hADSC pre-expanded with either hPL (hPL-hADSC) or FBS (FBS-hADSC) had an effect on neuronal regeneration, we performed a direct co-culture, where a single DRG explant was added on top of a sub-confluent layer of hADSC grown in media with the respective supplement. The DRG outgrowth, in terms of both maximal neurite length and axonal area, was significantly increased by the hPL-hADSC compared to hADSC grown in FBS-supplemented medium (FBS-hADSC) (*p* < 0.05, *p* < 0.001, Fig. 4g, h). Interestingly, when hADSC condition by growth with hPL supplemented media were shifted to a serum-free condition at the moment of DRG coculture, the supportive nature of these cells with respect to nerve regeneration was confirmed for hPL-hADSC but no improved outgrowth was recorded when the cells had been cultured with FBS supplemented media (*p* < 0.05, Fig. 4i). Consistently, the hPL supplemented media themselves did not directly increase axonal elongation but acted by proxy of pre-conditioning the hADSC.

To better understand the impact of the hADSC secretome on DRG sprouting, we realized an indirect co-culture by growing DRG in either hPL-hADSC or alternatively FBS-hADSC conditioned media.

The hPL-hADSC secretome was shown to have a statistically significant effect on the regeneration of the DRG neurons, by leading to an increased maximal neurite length and neurite extension area (*p* < 0.05, Fig. 4g, h).

Overall, the direct contact between the hPL-hADSC and the DRG supports higher neuronal regeneration as evidenced by their impact on neurite maximal length, neurite extension area, compared to single DRG growth alone in hPL-supplemented media (*p* < 0.01, *p* < 0.001, Fig. 4g, h) or hPL-hADSC conditioned medium (*p* < 0.05, Fig. 4g, h).

## Discussion

Peripheral nerve injuries often involve young and working-age adults leading to high personal and socioeconomic costs. Currently, the best practice for surgical repair consists of a tension-free direct nerve coaptation or, in case of a gap, an autologous nerve graft (usually the sural nerve) [26]. Unfortunately, particularly with extended lesions, a full recovery is rarely achieved, and donor site morbidity is relevant with scarring, sensory deficit, and potential neuroma formation as recognized complications [27, 28]. As the autologous nerve graft technique is far from optimal, the development of alternative approaches for the repair of peripheral nerves is essential. The use of primary Schwann cells (SCs) to improve nerve regeneration has been applied with success in a rodent model [29]. Despite showing regeneration enhancement, SCs have limited clinical applications; the culture of sufficient SCs in order to achieve optimal conditions for transplantation in nerve conduits is time-consuming and requires particular care for in vitro expansion and constant high level of GFs. Moreover, SCs are not easily accessible without nerve biopsy; requiring the sacrifice of an autologous nerve, with the related complications of sensory loss and pain at the harvesting site [30].

On the contrary, the ideal transplantable cell should be easily accessible, proliferate rapidly in culture, and successfully integrate into host tissue with immunological tolerance. Experimental studies in rats have shown that ADSC transplantation represents an alternative strategy to create a favorable environment for nerve regeneration [31, 32]. The growing applicability of hADSC has opened new frontiers to nerve regeneration, in line with the growing application of stem cell therapy in a wide range of medical fields [33].

When progressing towards clinical translation, patient safeguarding and optimum safety are mandatory. The risk of infection from animal-derived products is especially pertinent during this unique time in the history of the viral pandemic disease [34]. Clinical trials evaluating the potential of stem cell therapies are growing faster than research that investigates alternative, xenogeneic-free methods for cell isolation and culture [35–37]. Considering the therapeutic potential of hADSC, in particular for peripheral nerve repair, we established a complete xeno-free protocol for cell culture isolation and expansion.

The traditional process with both enzymatic digestion and centrifugation may lead to a higher volume of hADSC in a smaller amount of time, with the drawback on unresolved safety issues [9]. Conversely, an explant-based method where the hADSC migrates out of the tissue, adhering to tissue culture plastic surfaces could be a valid alternative according to GMP for hADSC isolation [10].

Our results showed no significant difference between cells that had been either enzymatic or mechanically isolated from adipose tissue regarding morphology, proliferative potential, and stemness of the hADSC.

Regarding the cell culture medium additive, traditionally, hADSC are expanded with FBS as a medium supplement which assures effective support in growth and maintenance, preserving their adhesion, stem cell properties, and supporting trilineage differentiation potential (osteogenic, adipogenic, and chondrogenic) of MSCs with a low cost [38, 39]. Unfortunately, when FBS is used in a translational setting, anaphylactoid reactions and the risk of transmission of zoonoses have been reported due to an incomplete knowledge of its composition and heterogeneity between samples [38]. Recent reports agree that hPL, which supports cell expansion to a higher degree, but without altering the stemness profile of the hADSC, could be introduced as an efficient and safe substitute for FBS [40–42].

From this perspective, we compared human ADSC cultures in D-MEM medium supplemented with 10% of FBS or alternatively with 5% of hPL in terms of morphological profile, immunophenotype, multilineage differentiation, and potential proliferation assessment.

According to the morphology, hADSC was significantly modified by the medium supplement during the cell expansion. Globally, the hADSC grown in hPL showed a more elongated, spindle-like shape as other recent studies reported in literature [43, 44].

In terms of expression of key stem cell markers such as CD73, CD90, and CD105, our findings were consistent with the current literature with the expression of these in more than 80% of the cells, but lack of expression of CD34 and CD45 markers by hADSC in the four conditions [18, 45, 46].

Similarly, adipogenic differentiation potential has been maintained and even enhanced in cultures with hPL compared to FBS supplemented ones. Probably, this more acquired Oil-red-O staining by hPL-hADSC, not in line with other previous studies, could be explained by the smaller morphology and higher proliferation rate which lead to a higher density of cells per field compare to the counterpart (FBS-hADSC).

Surprisingly, in contrast with the prevalent literature too, we found that while hPL-cultured cells easily evidenced an osteogenic differentiation potential, the counterparts expanded in FBS did not differentiate, despite testing multiple FBS batches. Indeed, it seemed that the wide variability between the sources of human adipose cells and FBS samples could reduce the predictability of hADSC differentiation [25].

Metabolic activity of hADSC expanded with hPL was enhanced when compared to FBS-cultured cells in alignment with their increased proliferation rate, which both are in accord with the current literature [47]. As previously stated, morphology, proliferation, and metabolic activity was not influenced significantly by the isolation methods.

Indeed, whether the native presence of neural GFs in the human platelet lysate could help enhance the neurotrophic effect of hPL-cultured hADSC (and not only change their shape into spindle-like) was a further investigation step in this study.

If the plethora of GFs contained in hPL could induce a sort of SC-like differentiation, we analyzed the expression of specific SC markers, such as nestin (indicating neural precursor cells), GFAP (mature astrocyte marker), MPZ (Schwann cell-specific marker), and STRO-1 [48]. Neither nestin nor GFAP could be detected in any of the samples, suggesting that hADSC were not committed to a SC-like phenotype as first described by Kingham et al. for differentiation via GFs [19]. On the contrary, some hPL-cultured hADSC showed a low level of MPZ. Conversely, STRO-1, a marker, which is expressed typically by MSCs prone to neural differentiation, was detected in hADSC cultured with either medium additives without a significant difference between these (Fig. 3c).

In order to assess SC-like commitment of hADSC cultured with hPL, we analyzed the GF secretion and specific gene expression in comparison with hADSC cultured in FBS. When we evaluated the level of GDNF, NGF, and BDNF in conditioned medium, collected after cell confluence in both culture condition (hPL and FBS), hPL-cultured cells secreted all GFs at a level significantly higher than that seen for FBS-cultured cells, suggesting that hPL could increase neurotrophic properties of hADSC.

Interestingly, levels of BDNF in hPL-conditioned medium were significantly lower than in hPL non-conditioned medium, suggesting that hADSC used the growth factors contained in hPL supplement. Indeed, the BDNF/NTRK2 axis is regulated by negative feedback (in line with our PCR results) and recent literature underlines its role in metabolic homeostasis both in neural and non-neural tissue (e.g., adipose tissue) [49]. Thus, the abundance of GF (e.g., BDNF) coming from hPL may inhibit the expression of NTRK2 in the hADSC to prevent an over-uptake and may suppress further production (in line with our ELISA results). Conversely, a lower detected level of NGF in hPL medium alone, but a higher one in hPL-conditioned medium, even if not significantly, could be the result of an increased hADSC secretion of the GF, in line with a similar expression profile of its receptors (*NGFR* and *NTRK1).*

These data, even if preliminary, suggest that hPL grown-hADSC modify their GF intake and their surface gene expression, and as an extent, may induce a potential release when transplanted in vivo.

When mimicking the in vivo neural microenvironment in functional co-cultures with DRG organotypic explants, the functional outcomes observed were the results of a combination of released factors and cell-to-cell interactions. The indirect co-culture method, applying the hADSC-conditioned medium only, showed how the hADSC-secretome [50] has a neurotrophic effect, with soluble factors released by the hADSC. Further, hADSC exerted an even stronger neurotrophic effect on primary neurons in the direct contact coculture model. This effect seemed mainly cell-interaction related, as non-conditioned medium, despite containing a relevant amount of growth factors, did not influence significantly neurite outgrowth. Similar outcomes of the enhanced neurotrophic potential of hADSC in response to HPL-conditioning were reported recently in a co-culture in vitro model with chicken embryonic DRG [51]. Whether such factors could be internalized by cells during culture [52] and potentially released or influencing hADSC gene/protein expression will be a matter of further in vitro studies of our group, together with in vivo applications.

## Conclusions

Our results greatly support the combination of the mechanical isolation process of the adipose tissue with the hPL supplement culture medium as a novel, bio-safe, and xenogeneic-free protocol to obtain hADSC ready for the human cell therapy use. hPL-cultured hADSC showed cell stability without altering their stem properties compared to classical FBS-supplemented hADSC, while they expressed a superior proliferation rate. In addition, regarding the applicability of the hADSC in peripheral nerve repair after injury, our findings suggest an important role of hPL in enhancing the neurogenic properties of the hADSC. These observations finally speculate that hADSC supported and expanded by hPL may provide an effective cell population and could be a clinically translatable route towards new methods of peripheral nerve repair.

## Data Availability

The datasets used and/or analyzed during the current study are available from the corresponding author on reasonable request and deposited at the University of Glasgow and Lausanne.

## Abbreviations

BDNF: Brain-derived growth factor
CD: Cluster of differentiation
DRG: Dorsal root ganglia
ECM: Extra-cellular matrix
FBS: Fetal bovine serum
GDNF: Glial cell line-derived neurotrophic factor
GMP: Good manufacturing practice
GFs: Growth factors
hADSC: Human adipose-derived stem cells
hPL: Human platelet lysate
MSCs: Mesenchymal stem cells
NGF: Nerve-derived growth factor
NTF3: Neurotrophin 3
PNI: Peripheral nerve injuries
SCs: Schwann cells
